# Evidence for Tethering of Human Cytomegalovirus Genomes to Host Chromosomes

**DOI:** 10.3389/fcimb.2020.577428

**Published:** 2020-09-30

**Authors:** Katrin Mauch-Mücke, Kathrin Schön, Christina Paulus, Michael M. Nevels

**Affiliations:** ^1^Institute for Medical Microbiology and Hygiene, University of Regensburg, Regensburg, Germany; ^2^Biomedical Sciences Research Complex, University of St Andrews, St Andrews, United Kingdom

**Keywords:** human herpesvirus, human cytomegalovirus, immediate-early 1, viral episome, genome maintenance, chromatin tethering, mitotic chromosome, fluorescence *in situ* hybridization

## Abstract

Tethering of viral genomes to host chromosomes has been recognized in a variety of DNA and RNA viruses. It can occur during both the productive cycle and latent infection and may impact viral genomes in manifold ways including their protection, localization, transcription, replication, integration, and segregation. Tethering is typically accomplished by dedicated viral proteins that simultaneously associate with both the viral genome and cellular chromatin via nucleic acid, histone and/or non-histone protein interactions. Some of the most prominent tethering proteins have been identified in DNA viruses establishing sustained latent infections, including members of the papillomaviruses and herpesviruses. Herpesvirus particles have linear genomes that circularize in infected cell nuclei and usually persist as extrachromosomal episomes. In several γ-herpesviruses, tethering facilitates the nuclear retention and faithful segregation of viral episomes during cell division, thus contributing to persistence of these viruses in the absence of infectious particle production. However, it has not been studied whether the genomes of human Cytomegalovirus (hCMV), the prototypical β-herpesvirus, are tethered to host chromosomes. Here we provide evidence by fluorescence *in situ* hybridization that hCMV genomes associate with the surface of human mitotic chromosomes following infection of both non-permissive myeloid and permissive fibroblast cells. This chromosome association occurs at lower frequency in the absence of the immediate-early 1 (IE1) proteins, which bind to histones and have been implicated in the maintenance of hCMV episomes. Our findings point to a mechanism of hCMV genome maintenance through mitosis and suggest a supporting but non-essential role of IE1 in this process.

## Introduction

Non-covalent tethering of viral genomes to host cell chromosomes is an intriguing phenomenon observed across an increasing number of DNA and RNA viruses (reviewed in Knipe et al., 2012; Aydin and Schelhaas, 2016; Chiu and Sugden, 2018; Poletti and Mavilio, 2018; De Leo et al., 2020). Sometimes the relevance of viral genome tethering has remained unclear, such as for adenoviruses (Puvion-Dutilleul and Pichard, 1992; Schröer et al., 1997). However, a unifying purpose of tethering appears to be persistent intranuclear infection. For some papillomaviruses and retroviruses, tethering serves to deliver viral genomes to the nucleus upon nuclear envelope breakdown during mitosis (Roe et al., 1993; Lewis and Emerman, 1994; Bieniasz et al., 1995; Day et al., 1998; Pyeon et al., 2009; Aydin et al., 2014). Tethering of pre-integration complexes (PICs) also determines the chromosomal integration sites of retrovirus genomes including those of Prototype Foamy Virus (PFV) (Tobaly-Tapiero et al., 2008; Lesbats et al., 2017; Lesbats and Parissi, 2018) and Human Immunodeficiency Virus 1 (Llano et al., 2006; Meehan et al., 2009). Papillomaviruses and herpesviruses do not generally link their DNA covalently to the host genome but usually persist as extrachromosomal episomes. These viruses often depend on episome tethering to mitotic chromosomes for segregation and partitioning of their genomes to daughter cell nuclei during latent infection when no infectious particles are produced (reviewed in Feeney and Parish, 2009; Knipe et al., 2012; Aydin and Schelhaas, 2016; Chiu and Sugden, 2018; De Leo et al., 2020). Remarkably, mitotic chromosome tethering for viral genome segregation extends to the Borna Disease Virus (BDV), a persistent non-reverse transcribing RNA virus (Tomonaga, 2007; Matsumoto et al., 2012; Hirai et al., 2016). In addition to securing nuclear localization and mitotic segregation, tethering has been implicated in defense evasion, epigenetic programming, transcriptional regulation, and replication dynamics of viral genomes during both latent and productive infections (reviewed in Knipe et al., 2012; Aydin and Schelhaas, 2016; Chiu and Sugden, 2018; Poletti and Mavilio, 2018; De Leo et al., 2020).

Human Cytomegalovirus (hCMV) is a member of the herpesvirus family, sub-classified as a β-herpesvirus. Various highly differentiated human cell types including primary fibroblasts are susceptible to hCMV infection and permissive for viral replication (reviewed in Sinzger et al., 2008a; Revello and Gerna, 2010; Li and Kamil, 2016; Nguyen and Kamil, 2018; Gerna et al., 2019). In contrast, hCMV establishes latency in a subset of poorly differentiated susceptible cells of the myeloid lineage including dividing hematopoietic progenitors (reviewed in Poole and Sinclair, 2015; Dupont and Reeves, 2016; Collins-McMillen et al., 2018; Nikitina et al., 2018; Forte et al., 2020). As in most herpesviruses, hCMV genomes are thought to be maintained as extrachromosomal, covalently closed circular episomes within latently infected cell nuclei (Bolovan-Fritts et al., 1999). In several human and animal γ-herpesviruses, the basic mechanisms of viral episome maintenance have been established. The two human γ-herpesviruses, Epstein-Barr Virus (EBV) and Kaposi's Sarcoma-Associated Herpesvirus (KSHV), both exhibit a chromosome tethering mechanism for episome maintenance through latency in dividing cells (reviewed in Chiu and Sugden, 2018; De Leo et al., 2020). In contrast, the latent genomes of two human β-herpesviruses, human herpesvirus 6A and 6B, appear to be maintained by chromosomal integration at telomeres rather than non-covalent tethering, although the latter has not been excluded (reviewed in Flamand, 2018; Aimola et al., 2020). For hCMV, it remains to be determined whether an episome tethering mechanism exists in latently infected cells. Likewise, it has not been studied whether hCMV genomes are tethered to host chromosomes during productive infection.

Viruses have evolved dedicated proteins to accomplish chromosome tethering. Human and bovine papillomavirus early 2 (E2) proteins, EBV nuclear antigen 1 (EBNA1), and KSHV latency-associated nuclear antigen (LANA) are structurally and functionally orthologous tethering proteins. They all simultaneously bind to the genome of their respective viruses and to host chromosomes via DNA, histone and/or non-histone chromatin protein interactions to confer latent episome tethering, segregation and maintenance through cell division (reviewed in Feeney and Parish, 2009; Knipe et al., 2012; Aydin and Schelhaas, 2016; Chiu and Sugden, 2018; De Leo et al., 2020). LANA exhibits a C-terminal DNA binding domain that interacts with the terminal repeats in the KSHV genome (Hellert et al., 2015; Grant et al., 2018). To associate with host chromatin, LANA contains N- and C-terminal chromosome binding sites, the latter of which interacts with methyl-CpG-binding protein 2 (MECP2) (Krithivas et al., 2002; Matsumura et al., 2010). The N-terminal amino acids 5 to 22 comprise the chromatin tethering domain (CTD) which binds to the acidic patch formed by histone H2A–H2B dimers on the surface of the nucleosome core particle (Barbera et al., 2006a,b). Notably, several viral proteins other than LANA are known or predicted to target host chromosomes through a mechanism involving the acidic patch on the nucleosome. A chromatin binding site in the nucleocapsid domain of the PFV group-specific antigen (GAG), which is a structural component of PICs, interacts with H2A–H2B at the acidic patch and is required for chromosome tethering as well as integration site selection of incoming subviral complexes (Tobaly-Tapiero et al., 2008; Lesbats et al., 2017; Lesbats and Parissi, 2018). Nucleosome targeting via core histones appears to be conserved in the murine leukemia virus GAG protein p12, which tethers viral PICs to mitotic chromosomes through a mechanism complemented by chromatin binding modules from PFV GAG, E2, and LANA (Elis et al., 2012; Wight et al., 2012, 2014; Schneider et al., 2013; Brzezinski et al., 2016a,b; Wanaguru and Bishop, 2018; Wanaguru et al., 2018). Furthermore, the BDV ribonucleoprotein tethers the viral genome to host chromosomes and binds to core histones with a preference for H2A and H2B (Matsumoto et al., 2012). Finally, hCMV IE1 associates with mitotic chromatin via a 16 amino acid CTD located at the C-terminus (Lafemina et al., 1989; Wilkinson et al., 1998; Reinhardt et al., 2005). A ten amino acid nucleosome binding motif within the IE1 CTD interacts with H2A–H2B to target the acidic patch on the nucleosome in a way similar but not identical to LANA (Mücke et al., 2013; Fang et al., 2016). The IE1 CTD appears to be dispensable for hCMV replication in fibroblasts (Reinhardt et al., 2005; Shin et al., 2012; Mücke et al., 2013). However, a CTD containing smaller isoform of IE1 referred to as IE1 exon 4 has been proposed to mediate replication and maintenance of hCMV episomes in latently infected hematopoietic progenitor cells by binding to the terminal repeats in the viral genome (Tarrant-Elorza et al., 2014). These findings strongly suggest that IE1 might be a tethering protein of hCMV.

Here we investigate tethering of hCMV genomes to mitotic chromosomes in infected myeloid and fibroblast cells and a potential role of IE1 in this process.

## Methods

### Cells and Viruses

All cell cultures were kept at 37°C in a humidified atmosphere containing 5% CO_2_. MRC-5 human primary embryonic lung fibroblasts (Jacobs et al., 1970), obtained from the American Type Culture Collection (CCL-171), were maintained in Dulbecco's modified Eagle's medium (4.5 g/l glucose, 2 mM glutamine, 3.7 g/l sodium hydrogen carbonate, 1 mM sodium pyruvate) with 10% fetal calf serum, 100 U/ml penicillin and 100 μg/ml streptomycin. The human bone marrow-derived myelogenous leukemia cell line KG-1 (Koeffler and Golde, 1978), obtained from John Sinclair (University of Cambridge), was maintained in Roswell Park Memorial Institute 1,640 medium (4.5 g/l glucose, 2 mM L-glutamine, 2 g/l sodium hydrogen carbonate, 1 mM sodium pyruvate) with 10% fetal calf serum, 100 U/ml penicillin and 100 μg/ml streptomycin. Cells were regularly screened for mycoplasma contamination using a PCR assay (Uphoff and Drexler, 2014).

The low passage hCMV strain TB40E, reconstituted from bacterial artificial chromosome (BAC) clone TB40-BAC4 (Sinzger et al., 2008b), served as a wild-type virus (TBwt). Mutant derivatives of TBwt expressing either no IE1 (TBdlIE1) or IE1 with amino acids 476–491 deleted (TBIE1_1−475_) have been described (Mücke et al., 2013; Zalckvar et al., 2013). Cell-free virus stocks were produced upon electroporation of MRC-5 cells with TBwt or TBIE1_1−475_ BAC DNA, or MRC-5-derived TetR-IE1 cells (Knoblach et al., 2011; Harwardt et al., 2016) with TBdlIE1 BAC DNA. Titers were calculated by qPCR-based absolute quantification of intracellular viral genome copies following infection of MRC-5 cells as described (Krauss et al., 2009; Knoblach et al., 2011).

### Protein Analyses

Preparation of whole cell extracts, gel electrophoresis, immunoblotting, and immunofluorescence were performed as described (Mücke et al., 2013). The following antibodies were used for protein detection: rabbit anti-glyceraldehyde-3-phosphate dehydrogenase (GAPDH) (ab9485, Abcam), mouse anti-IE1 [ab30924 (IE1.G10), Abcam; 6E1, Santa Cruz], mouse anti-IE1/IE2 (MAB810R, Merck Millipore), horseradish peroxidase-conjugated goat anti-mouse (115-036-003, Dianova) or anti-rabbit (AP156P, Merck Millipore) and Alexa Fluor 488-conjugated goat anti-mouse (A-11001, Thermo Fisher).

### Fluorescence *in situ* Hybridization (FISH)

For hybridization probe preparation, 10 μg purified TB40-BAC4 DNA was linearized by *Pac*I digestion in the presence of 5 μg/ml RNase A (Sigma Aldrich). The DNA was extracted with phenol-chloroform-isoamyl alcohol using Phase Lock Gel Heavy tubes (Eppendorf) and precipitated overnight at −20°C with 2.5 volumes ≥99.8% ethanol, 0.01 volumes 3 M sodium acetate (pH 5.2) and 20 μg glycogen. The DNA was centrifuged (20,000 × g, 30 min, 4°C), washed twice in 1 ml 70% ethanol, air-dried and dissolved in ultrapure water. Probe labeling with ChromaTide Alexa Fluor 488-5-dUTP (Thermo Fisher) by nick translation was conducted under light protection following previously published protocols (Cremer et al., 2008; Solovei and Cremer, 2010). Briefly, a 50-μl reaction mixture containing 1 μg linearized BAC DNA, 5 μl 40 U/ml DNase I (New England Biolabs), 1.5 μl 10,000 U/ml DNA polymerase I *(E. coli)* (New England Biolabs), 1.5 μl 1 mM ChromaTide Alexa Fluor 488-5-dUTP, 4 μl 0.5 mM dATP/dCTP/dGTP (New England Biolabs), 5 μl 0.1 mM dTTP (New England Biolabs), 5 μl 0.1 M dithiothreitol and 5 μl 10 × nick translation buffer [500 mM Tris/HCl (pH 7.5), 50 mM MgCl_2_, 0.5 mg/ml bovine serum albumin] was prepared and incubated for 2 h at 15°C. The reaction was stopped by adding 1 μl 0.5 M ethylenediaminetetraacetic acid and heating for 10 min at 65°C. Unlabeled nucleotides were removed using Mini Quick Spin Columns (Sigma Aldrich) according to the manufacturer's instructions. Following overnight precipitation at −20°C with 2.5 volumes ≥99.8% ethanol, 10 μg Cot-1 DNA (Thermo Fisher), and 20 μg salmon sperm DNA (Thermo Fisher), the DNA was centrifuged (20,000 × g, 30 min, 4°C), washed twice in 1 ml 70% ethanol, air-dried, and dissolved in 12.5 μl formamide (Merck Millipore) under shaking at 37°C. Finally, one volume of a 20% dextran sulfate solution in 4 × saline-sodium-citrate (SSC) (1 × SSC: 15 mM sodium citrate, 150 mM NaCl) was added, and shaking was continued for 10 min at 37°C to facilitate complete dissolution. Before use, sodium dodecyl sulfate (SDS) was added to 0.1% for increased stringency, and the hybridization mix was centrifuged (20,000 × g, 10 min) to remove undissolved material.

For KG-1 infections, 5 × 10^6^ cells were collected by centrifugation (300 × g, 10 min) and mock-infected or infected with TBwt at a multiplicity of three in the presence of 30 μg/ml polybrene and with centrifugal enhancement (1,200 × g, 37°C, 2 h). After that, cells were subjected to a brief wash in phosphate-buffered saline (PBS), a 1-min wash in citrate buffer [40 mM citric acid/sodium citrate (pH 3.0), 10 mM KCl, 135 mM NaCl] and another brief PBS wash to remove extracellular virus particles. A second round of infection under the same conditions was conducted 24 h after the first infection. For MRC-5 infections, (nearly) confluent cells were mock-infected or infected with TBwt, TBdlIE1, or TBIE1_1−475_ at a multiplicity of three for 2 h before the inoculum was removed.

For preparation of mitotic chromosome spreads, cultures were maintained overnight in fresh medium with 0.025 μg/ml N-desacetyl-N-methylocolchicine (KaryoMAX Colcemid Solution in PBS, Thermo Fisher) to increase the number of mitotic cells. Culture supernatants were removed and collected, cells were trypsinized and combined with the correspondent supernatants, and samples were centrifuged (250 × g, 10 min). Following a brief wash in PBS and careful resuspension, cells were subjected to hypotonic treatment and fixation according to published protocols (Heller et al., 1995; Schröer et al., 1997). Cells suspended in fixative (3:1 methanol/acetic acid) were splashed onto precleaned glass slides using a Pasteur pipet. Samples were air-dried and subjected to digestion with 10 μg/ml RNase A in 2 × SSC for 1 h, three 5-min washes in 2 × SSC and one 2-min wash in 0.01 M HCl. After that, samples were incubated for 15 min in a fresh pepsin solution (0.005% in 0.01 HCl) at 37°C. This was followed by a brief wash in double-distilled water, two washes in 2 × SSC and consecutive 10-min washes in 70, 80, 90, and ≥99.8% ethanol. Finally, slides were dried at 55°C.

The hybridization reaction was started by carefully dropping 22 μl hybridization mix onto each slide. Coverslips were subsequently applied to the slides and sealed with Fixogum (Marabu). Samples were subjected to denaturation for 3 min at 75°C, and to hybridization overnight at 37°C. After that, slides were incubated in 2 × SSC until the coverslips came off. These steps were followed by three 5-min washes in formamide solution [50% in 2 × SSC (pH 7.0)] at 42°C, five 2-min washes in 2 × SSC at 42°C, one 2-min wash in Tween 20 solution (0.1% in 4 × SSC) at 42°C and one brief wash in PBS. Then, 4′,6-diamidino-2-phenylindole (DAPI) solution (0.33 μg/ml in PBS) was added for 15 min. Following a brief wash in PBS, nuclei were again treated with consecutive 70, 80, 90, and ≥99.8% ethanol washes and dried at 55°C. Finally, slides were mounted using SlowFade Gold (Thermo Fisher). Images were acquired using a Leica TCS SP8 confocal microscope equipped with a 405 nm diode laser/photomultiplier tube detector (DAPI) and an argon laser/hybrid detector (Alexa Fluor 488). Leica Application Suite Advanced Fluorescence 3.1.0 and Adobe Photoshop CS6 software were used for image processing. *P*-values were calculated based on unpaired, two-tailed Student's *t*-tests assuming unequal variances.

## Results

### hCMV Genomes Colocalize With Host Chromosomes in KG-1 Cells

**Figure 1:**
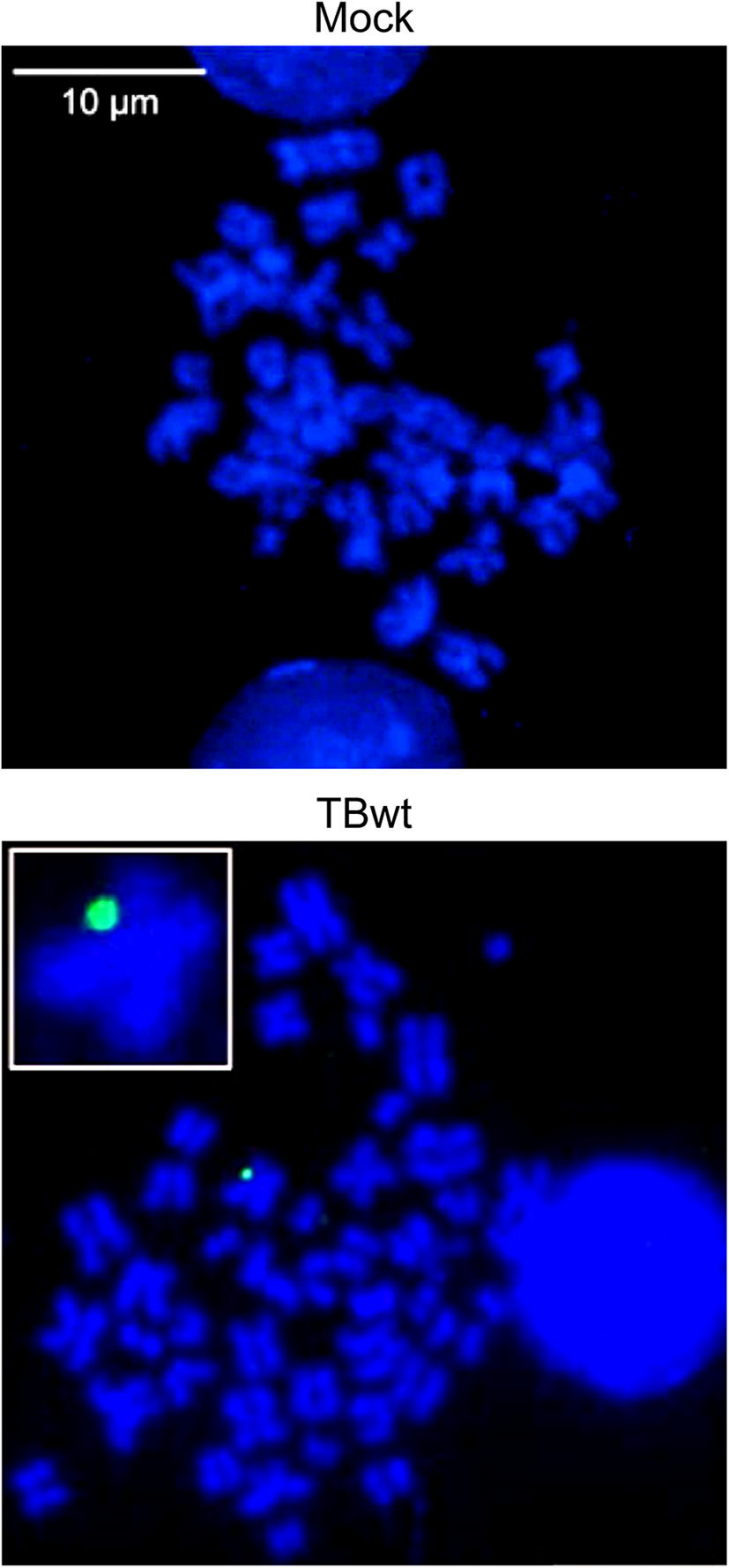
hCMV genome association with host mitotic chromosomes in myeloid cells. KG-1 cells were mock-infected or infected with TBwt. After three days following infection, metaphase spreads were prepared and subjected to FISH using an hCMV-specific probe. Representative confocal images (maximum projections from *z*-stacks of 0.3 μm slices) of mitotic chromosomes (blue) and hCMV genomes (green) are shown. The insert shows a magnified cellular chromosome with tethered hCMV genome. More images are provided in Supplementary Figure 1A.

A body of work has established that CD34+ hematopoietic progenitors and cells committed to the myeloid lineage serve as a natural reservoir of latent hCMV (reviewed in Poole and Sinclair, 2015; Dupont and Reeves, 2016; Collins-McMillen et al., 2018; Nikitina et al., 2018; Forte et al., 2020). Aspects of hCMV latency have been modeled using the CD34+ myeloblastic cell line KG-1 (Sindre et al., 2000; Poole et al., 2011; Albright and Kalejta, 2013). KG-1 cells remain CD34+ in culture, permit hCMV entry, maintain viral DNA over time but may not support infectious particle production (Albright and Kalejta, 2013). We infected KG-1 cells with hCMV (TB40E) at high multiplicity and visualized the localization of intracellular viral DNA in spatial relation to human mitotic (metaphase) chromosomes by FISH and confocal microscopy. The post-infection time points chosen in this experiment were 3 and 9 days, based on previous observations indicating delayed nuclear translocation of hCMV genomes in myeloid cells (Kim et al., 2016) and maintenance of viral DNA for at least 10 days in KG-1 cells (Albright and Kalejta, 2013). Virus-specific signals were detected in 2.5% of analyzed interphase nuclei (*n* = 1299) at 3 days post infection. At 9 days post infection, virus-specific signals were detected in 0.7% of analyzed interphase nuclei (*n* = 1514). Single fluorescence signals, each likely corresponding to one individual viral genome, were detected at the periphery of metaphase chromosomes in hCMV-infected but not in mock-infected mitotic cells (Figure 1 and Supplementary Figure 1A). The colocalized viral genomes are presumably linked physically to host chromosomes, since untethered episomes should be washed away when cells subjected to hypotonic treatment are splashed onto glass slides for metaphase spread preparation and by the stringent washes after hybridization required for signal specificity. The extremely low frequency of KG-1 cells that were both mitotic and infected precluded a quantitative or comparative analysis of viral genome tethering in this system. Nonetheless, these results provide initial evidence for the idea that hCMV episomes may be tethered to host mitotic chromatin during latent infection of human myeloid cells.

### hCMV Genomes Colocalize With Host Chromosomes in MRC-5 Cells

**Figure 2:**
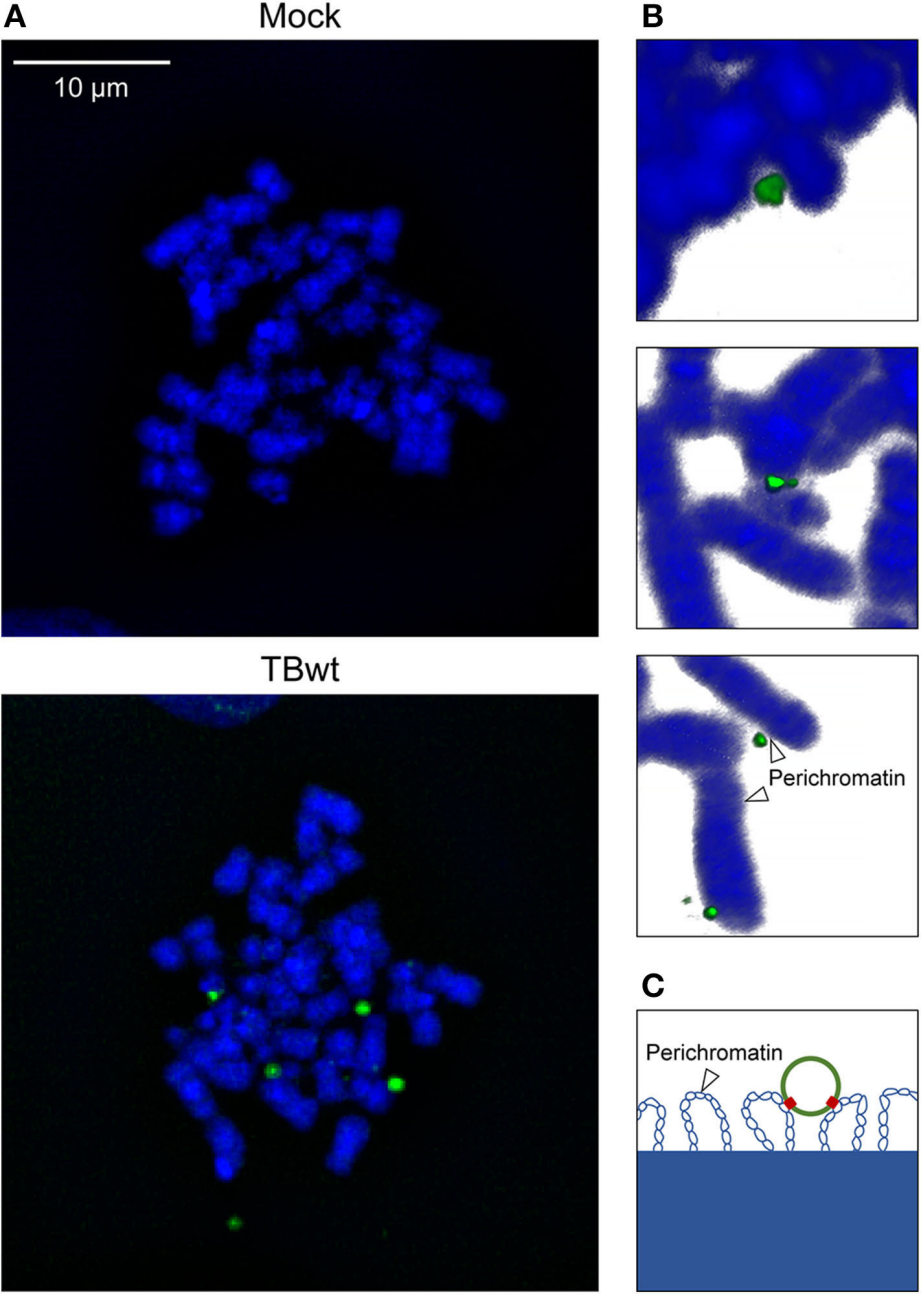
hCMV genome association with host mitotic chromosomes in fibroblasts. MRC-5 cells were mock-infected or infected with TBwt. After three days following infection, metaphase spreads were prepared, and subjected to FISH using an hCMV-specific probe. **(A)** Representative confocal images (maximum projections from *z*-stacks of 0.3 μm slices) of mitotic chromosomes (blue) and hCMV genomes (green) are shown. More images are provided in Supplementary Figure 1B. **(B)** Representative three-dimensional reconstructions of magnified cellular chromosomes with hCMV genomes attached to perichromatin. **(C)** Illustration of a viral episome associated with perichromatin loops extending from condensed chromatin. Histones are not depicted. Blue, host DNA; green, viral episome; red, tethering proteins.

MRC-5 fibroblasts are highly permissive to hCMV replication and have been widely used to study the viral productive cycle. MRC-5 cells infected with hCMV at high multiplicity were subjected to FISH and confocal microscopy. One to eight virus-specific signals were detected in 94% of analyzed interphase nuclei (*n* = 100) at 18 h post infection. Likewise, individual or multiple (up to 9) fluorescent signals were detected in mitotic cells, most of them associated with the periphery of metaphase chromosomes referred to as “perichromatin” (Figures 2A,B and Supplementary Figure 1B). Perichromatin exhibits a less compact structure with loops extending outwards from the chromosome surface (Figure 2C) and is not as efficiently stained with DAPI compared to the highly condensed cores of mitotic chromosomes (reviewed in Cremer et al., 2004; Fakan and Van Driel, 2007; Cremer et al., 2015; Masiello et al., 2018; Cremer et al., 2020). Thus, even colocalized viral genomes that appear discrete from DAPI-stained host chromatin are likely to be physically attached (Figures 2B,C). These findings suggest that hCMV episomes may be tethered to host mitotic chromatin during productive infection of human fibroblasts.

### hCMV IE1 Supports Viral Genome Colocalization With Host Chromosomes

**Figure 3:**
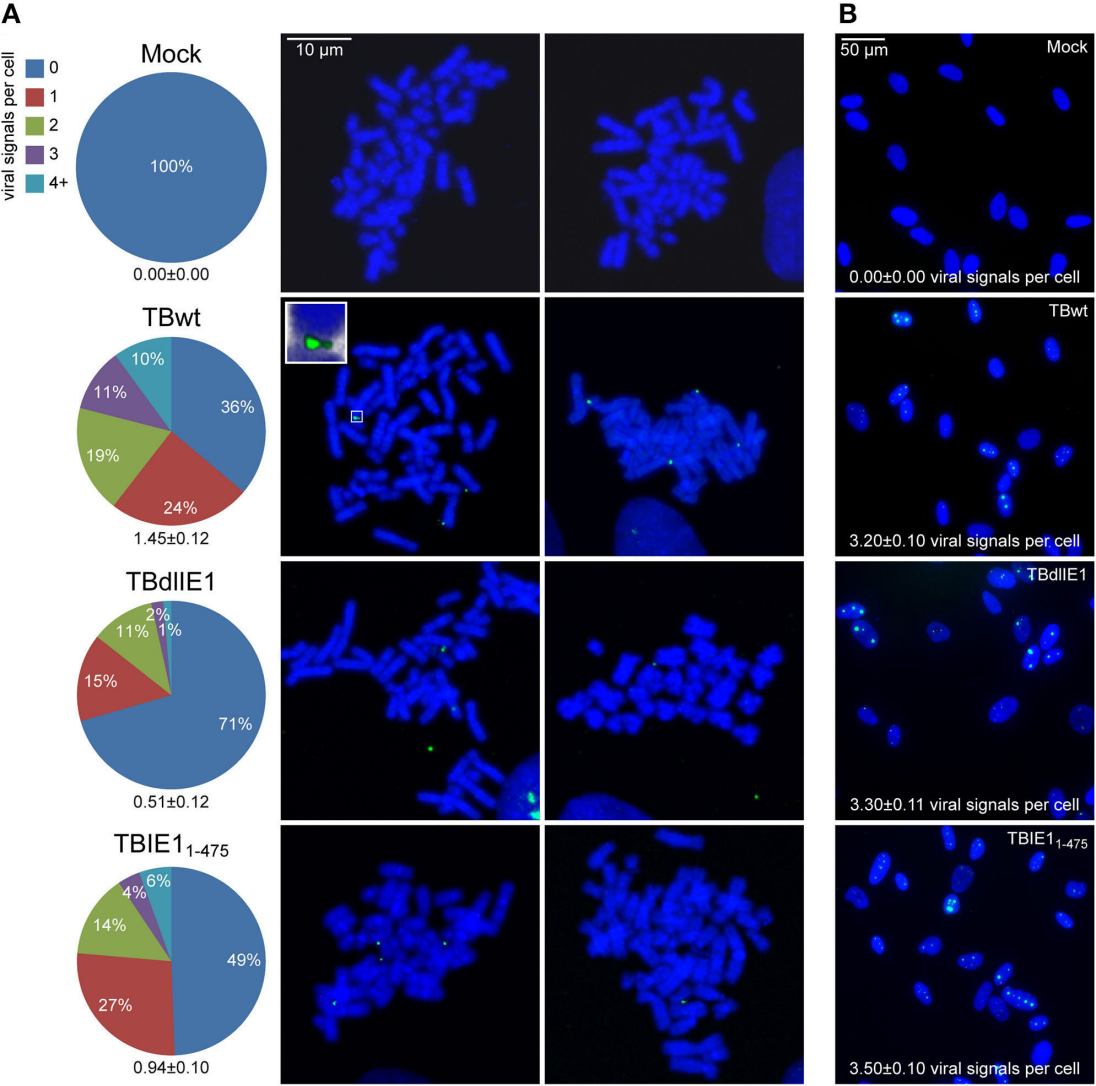
hCMV IE1 and the IE1 CTD promote viral genome association with host mitotic chromosomes. MRC-5 cells were mock-infected or infected with hCMV (TBwt and TBIE1_1−475_) for 18 or 42 h (TBdlIE1). **(A)** Metaphase spreads were prepared and subjected to FISH using an hCMV-specific probe. The number of hCMV-specific signals colocalized with human chromosomes per metaphase spread from 100 (mock), 119 (TBwt), 146 (TBdlIE1), or 182 (TBIE1_1−475_) mitotic cells was counted, and results are presented as percentage of spreads with 0–≥4 signals and as average number of signals per cell +/- standard error of the mean (numbers below pie charts). Two representative confocal images (maximum projections from *z*-stacks of 0.3 μm slices) of mitotic chromosomes (blue) and hCMV genomes (green) from each infection are shown. The insert shows a magnified cellular chromosome tip with attached hCMV genomes from a three-dimensional reconstruction. More images are provided in Supplementary Figure 1B. **(B)** Representative confocal images of interphase nuclei from the infections in (A) are shown. hCMV-specific signals counted from 100 interphase nuclei of each infection are represented as average number of signals per cell +/- standard error of the mean.

The association of viral episomes with host chromosomes is usually mediated by virus-encoded tethering proteins (reviewed in Feeney and Parish, 2009; Knipe et al., 2012; Aydin and Schelhaas, 2016; Chiu and Sugden, 2018; De Leo et al., 2020). Previous work has shown that the hCMV IE1 protein shares features with known tethering proteins as it binds to both viral and mitotic cellular chromatin (Lafemina et al., 1989; Tarrant-Elorza et al., 2014). Host chromosome association depends on a nucleosome binding motif in the IE1 CTD (amino acids 476–491) (Reinhardt et al., 2005; Mücke et al., 2013; Fang et al., 2016). To study whether IE1 contributes to hCMV genome tethering, FISH and confocal microscopy were conducted on mitotic and interphase MRC-5 cells inoculated with equal infectious genome equivalents of a wild-type strain (TBwt) and two previously characterized mutant derivatives (TBdlIE1 and TBIE1_1−475_). TBdlIE1 is selectively deficient for IE1 expression, and TBIE1_1−475_ encodes a CTD-deleted IE1 (Mücke et al., 2013; Zalckvar et al., 2013). IE1_1−475_ accumulates with kinetics and to levels comparable to the wild-type protein in hCMV-infected fibroblasts (Supplementary Figure 2A) (Shin et al., 2012; Mücke et al., 2013). However, unlike full-length IE1, the truncated protein is unable to associate with mitotic chromatin (Supplementary Figure 2B) (Shin et al., 2012; Mücke et al., 2013). Viral genomes linked to the periphery of metaphase chromosomes were observed following infection with all three recombinant viruses. However, fewer chromosome preparations carried at least one colocalized viral genome in infections with TBdlIE1 (29%) compared to TBwt (64%). The difference between TBdlIE1 and TBwt was particularly striking with regards to three or more colocalized viral genomes per cell (3% compared to 21%, respectively). We also observed a larger proportion of viral genomes outside the proximity of host chromosomes in metaphase spreads derived from TBdlIE1 compared to TBwt infections. The average number of colocalized viral genomes per chromosome spread was roughly 3-fold lower in TBdlIE1 compared to TBwt infections (*p* < 0.001) (Figure 3A). A similar but less pronounced phenotype was observed in two experiments with TBIE1_1−475_. This mutant exhibited a less than 2-fold difference in the average number of colocalized viral genomes compared to both TBwt (*p* ≤ 0.01) and TBdlIE1 (*p* < 0.001) (Figure 3A and Supplementary Figure 3). The differences in chromosome association between TBwt, TBdlIE1, and TBIE1_1−475_ were not due to varying intracellular viral genome copies as documented by very similar numbers of virus-specific signals in interphase cells infected with the three recombinant viruses (Figure 3B).

These results demonstrate that IE1 is not required for the association of hCMV genomes with host chromosomes. However, IE1 appears to serve a supportive role in putative viral episome tethering via both CTD-dependent and CTD-independent mechanisms.

## Discussion

The results from this study demonstrate for the first time that hCMV genomes associate with host chromosomes in both non-permissive KG-1 as well as permissive MRC-5 cells. We have not examined whether our findings extend to cell types beyond those two and to virus strains other than TB40E. However, it would not be surprising if the observations from this initial study were more generally relevant. Our results are suggestive of a tethering mechanism that supports the maintenance of at least some hCMV episomes during latent infection and may also benefit the viral productive cycle.

FISH on mitotic chromosome spreads is considered the gold standard to examine viral episome tethering (reviewed in Feeney and Parish, 2009; Knipe et al., 2012; Aydin and Schelhaas, 2016; Chiu and Sugden, 2018; De Leo et al., 2020). The significant shearing forces during chromosome spreading and the stringent washing steps following hybridization should remove untethered viral genomes from the FISH samples. Thus, almost all viral signals observed on the metaphase spreads should correspond to hCMV genomes linked physically to host chromosomes. Nonetheless, we are aware that FISH does not formally demonstrate this physical interaction. To our knowledge, there is no established biochemical method that could be used to confirm a physical association between mitotic chromosomes and viral episomes. Chromosome conformation capture (3C) methods would be an option to examine interactions between viral and cellular chromatin in interphase cells, especially if the interacting DNA sequences were known. However, 3C on rare populations of infected mitotic cells may not be feasible.

Interestingly, hCMV genomes appear to localize exclusively to the periphery of human chromosomes. Targeting of viral episomes to the chromosome surface referred to as perichromatin was previously reported in latent EBV and KSHV infections (Deutsch et al., 2010; Rahayu et al., 2016). Perichromatin spreads along the interface between highly condensed chromatin domains, which may be inaccessible for larger macromolecular aggregates, and interchromatin channels. It is characterized by a less condensed structure and functional importance as a major site of chromatin modification, transcriptional activity, DNA replication, and DNA repair (reviewed in Cremer et al., 2004; Fakan and Van Driel, 2007; Cremer et al., 2015; Masiello et al., 2018; Cremer et al., 2020). The nuclear localization and perichromatin association conferred by chromosome tethering protects viral genomes from cytoplasmic sensing and provides opportunities for epigenetic programming, transcriptional regulation as well as DNA replication and/or segregation.

**Figure 4:**
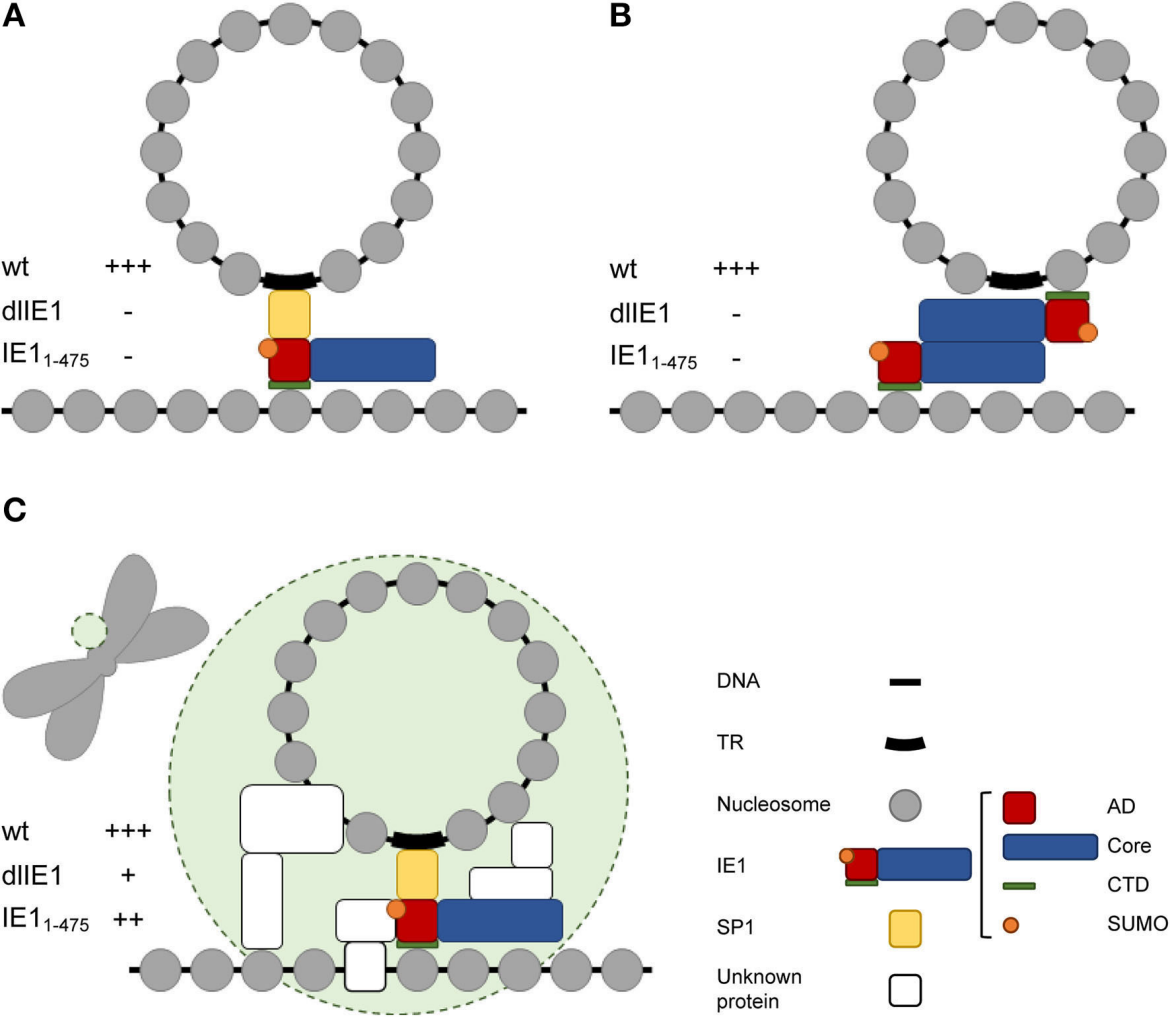
Models of IE1 function in hCMV genome tethering. **(A)** Model proposed by Tarrant-Elorza et al. (2014) based on interactions of IE1 with Sp1 and H2A–H2B. **(B)** Alternative model based on interactions of IE1 with itself and H2A–H2B. **(C)** Hypothetical hCMV “episome body” (green circle) composed of IE1 and multiple unknown proteins that might include the IE1 interacting proteins PML, STAT2, and/or STAT3 and chromatin-associated hCMV proteins other than IE1. The tethering phenotype of wild-type and mutant viruses predicted for each model is indicated by +++ (full activity), ++ (slightly reduced activity), + (severely reduced activity), – (no activity). Note that only the model shown in (C) is compatible with the results presented in this study. AD, acidic domain; Core, core domain; SUMO, small ubiquitin-like modifier; TR, terminal repeats.

It has been proposed that a short IE1 isoform referred to as IE1 exon 4 may serve as a tethering protein for hCMV episomes (Tarrant-Elorza et al., 2014). IE1 exon 4 facilitates hCMV genome replication and maintenance in latently infected cells by a mechanism that depends on both the acidic domain and the CTD (Figure 4A). In contrast to other viral tethering proteins including E2, EBNA1, and LANA, there is no evidence for a DNA binding domain in IE1. Instead, the acidic domain in IE1 appears to associate with the viral genome indirectly via the cellular transcription factor SP1 which binds to the terminal repeats, while the CTD interacts with the acidic patch formed by H2A–H2B on the nucleosome surface (Mücke et al., 2013; Tarrant-Elorza et al., 2014; Fang et al., 2016). It has also been shown that IE1 forms homodimers (Scherer et al., 2014; Klingl et al., 2015). Thus, simultaneous binding to both histones in viral as well as cellular chromatin to mediate tethering appears feasible, not least because hCMV genomes form nucleosomes in infected cell nuclei (Figure 4B) (Nitzsche et al., 2008; Zalckvar et al., 2013).

To our surprise, the genomes of a mutant virus (TBdlIE1) that neither expresses canonical IE1 nor any of its smaller isoforms still localized to chromosomes. However, chromosome association was less efficient in the absence of IE1 compared to wild-type virus infections. A mutant virus expressing CTD-deleted IE1 (TBIE1_1−475_) exhibited an even smaller colocalization defect compared to TBdlIE1. Although unlikely, we cannot rule out that these mutant phenotypes result from deletion of viral DNA sequences that might be relevant to host chromosome association irrespective of IE1 protein function. More likely, IE1 exhibits a supporting role in putative viral genome tethering with contributions from both CTD-dependent and CTD-independent mechanisms. The latter may involve cellular proteins that IE1 recruits to mitotic chromatin including PML, STAT2 or STAT3 (Paulus et al., 2006, 2020; Huh et al., 2008; Krauss et al., 2009; Dimitropoulou et al., 2010; Shin et al., 2012; Reitsma et al., 2013; Harwardt et al., 2016). Our results are consistent with complex and partly redundant tethering mechanisms for hCMV episomes. Although highly speculative at this point, we envision that an hCMV “episome body” might exist that combines several proteins to recruit viral genomes to host chromosomes, stabilize viral episome complexes, and program viral chromatin for transcription and replication (Figure 4C).

## Data Availability Statement

All datasets generated for this study are included in the article/Supplementary Material.

## Author Contributions

CP and MN designed the study. KM-M, KS, and CP performed the experiments and analyses. MN wrote the manuscript. All authors contributed to the article and approved the submitted version.

## Conflict of Interest

The authors declare that the research was conducted in the absence of any commercial or financial relationships that could be construed as a potential conflict of interest.
