# Supplementary figures and images for: Evidence for Tethering of Human Cytomegalovirus Genomes to Host Chromosomes
Source: Front Cell Infect Microbiol. 2020 Sep 30;10:577428. doi: 10.3389/fcimb.2020.577428 (PMC7561393; doi:10.3389/fcimb.2020.577428)

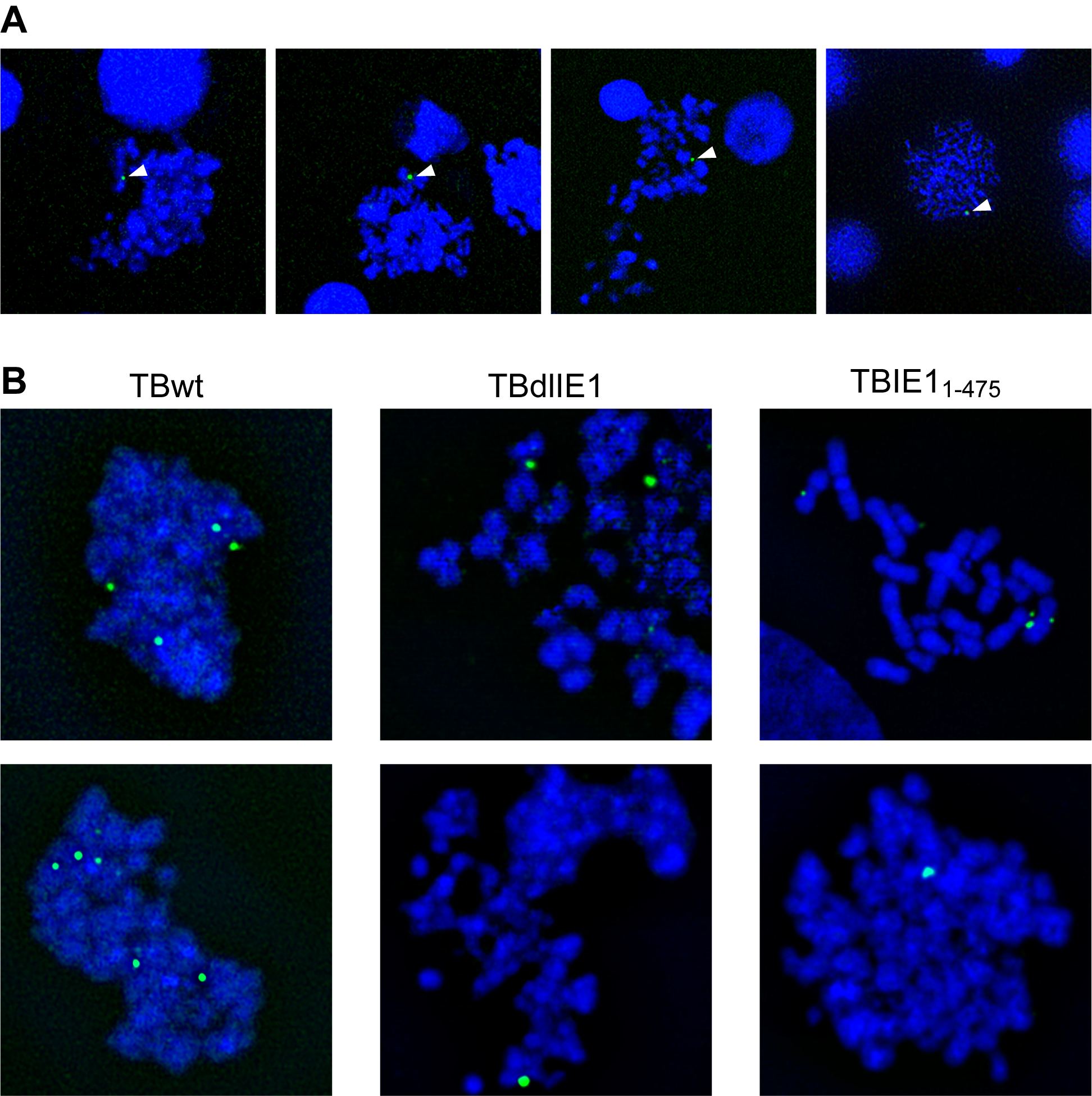

Supplement: Supplementary file 1 [file Image_1.TIF]

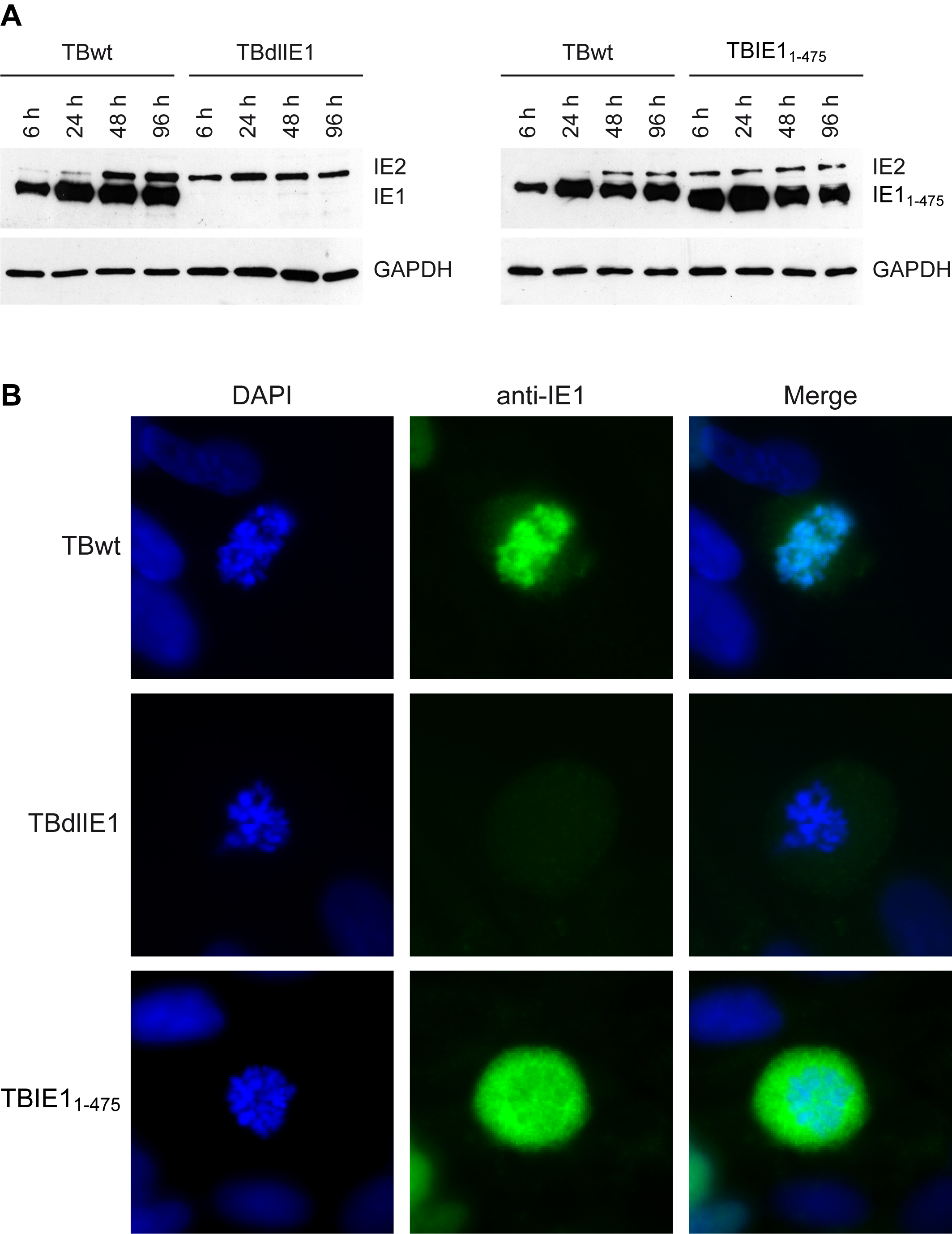

Supplement: Supplementary file 2 [file Image_2.TIF]

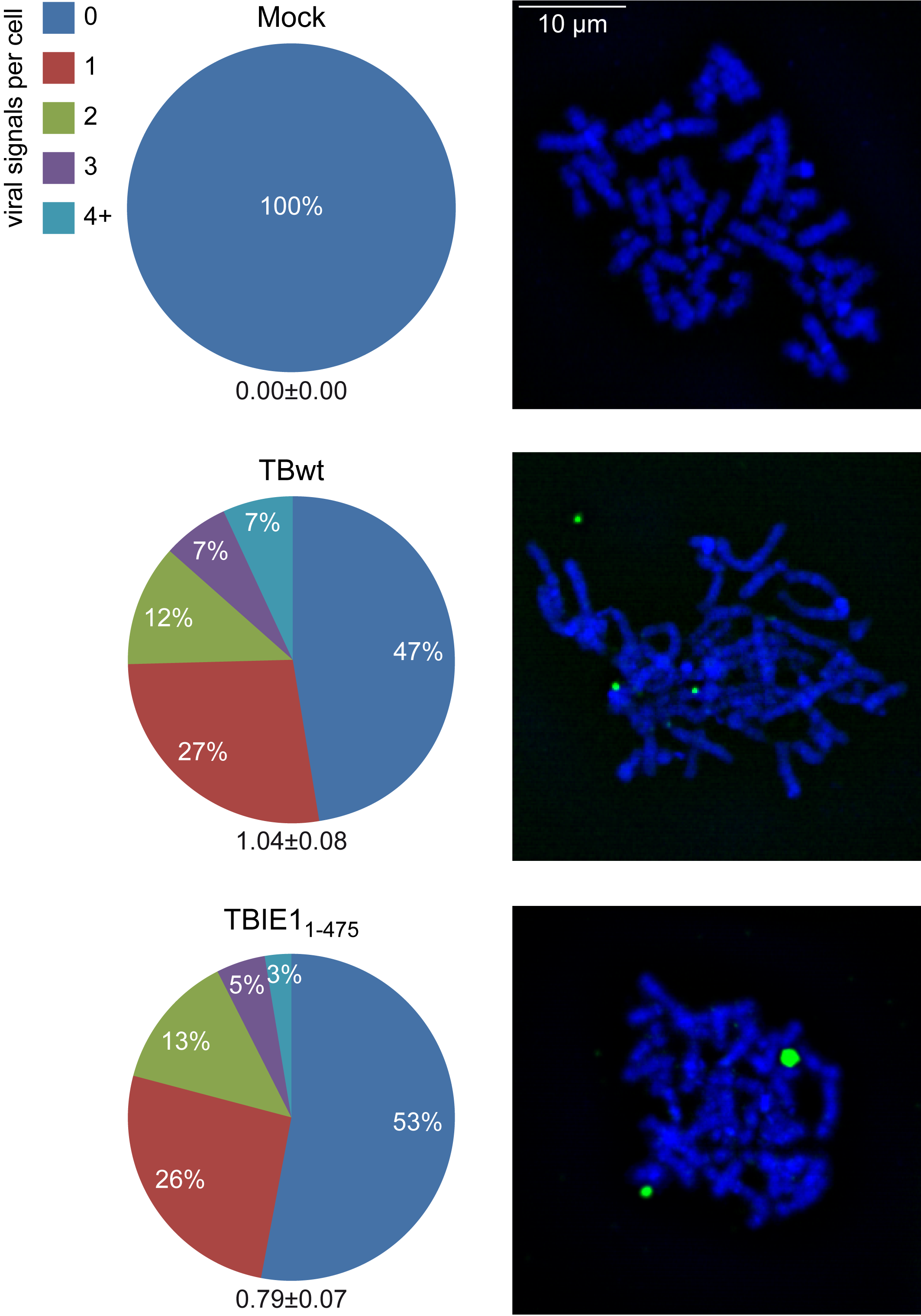

Supplement: Supplementary file 3 [file Image_3.TIF]
